# Biochemical characterization of a psychrophilic and halotolerant α–carbonic anhydrase from a deep-sea bacterium, *Photobacterium profundum*

**DOI:** 10.3934/microbiol.2023028

**Published:** 2023-06-19

**Authors:** Vijayakumar Somalinga, Emily Foss, Amy M. Grunden

**Affiliations:** 1 Department of Biological & Biomedical Sciences, Southwestern Oklahoma State University, 100 Campus Drive, Weatherford, OK 73096, USA; 2 Department of Plant and Microbial Biology, North Carolina State University, 4550A Thomas Hall, Campus Box 7612, Raleigh, NC 27695, USA

**Keywords:** *Photobacterium profundum*, α-carbonic anhydrase, halotolerance, biomineralization, carbon capture

## Abstract

Prokaryotic α–carbonic anhydrases (α-CA) are metalloenzymes that catalyze the reversible hydration of CO_2_ to bicarbonate and proton. We had reported the first crystal structure of a pyschrohalophilic α–CA from a deep-sea bacterium, *Photobacterium profundum* SS9. In this manuscript, we report the first biochemical characterization of *P. profundum* α–CA (PprCA) which revealed several catalytic properties that are atypical for this class of CA's. Purified PprCA exhibited maximal catalytic activity at psychrophilic temperatures with substantial decrease in activity at mesophilic and thermophilic range. Similar to other α–CA's, Ppr9A showed peak activity at alkaline pH (pH 11), although, PprCA retained 88% of its activity even at acidic pH (pH 5). Exposing PprCA to varying concentrations of oxidizing and reducing agents revealed that N-terminal cysteine residues in PprCA may play a role in the structural stability of the enzyme. Although inefficient in CO_2_ hydration activity under mesophilic and thermophilic temperatures, PprCA exhibited salt-dependent thermotolerance and catalytic activity under extreme halophilic conditions. Similar to other well-characterized α–CA's, PprCA is also inhibited by monovalent anions even at low concentrations. Finally, we demonstrate that PprCA accelerates CO_2_ biomineralization to calcium carbonate under alkaline conditions.

## 1. Introduction

Rapid industrialization starting from mid-eighteenth century has steadily contributed to the dramatic increase of greenhouse gases in our atmosphere. Global population growth and the resulting anthropogenic activities such as energy production, energy consumption, industrial manufacturing, vehicle emissions, etc. has added to increased CO_2_ release into our atmosphere which has disrupted our natural carbon sequestration processes, culminating in climate change. Some of the biggest contributors to global CO_2_ emissions include power plants and other industrial processes that use fossil fuel combustion for energy. For example, in 2018 alone nearly 55.3 GtCO_2_e (Gigatons of carbon dioxide emissions) were released into our atmosphere [1], of this 55.3 GtCO_2_e, about 37.5 GtCO_2_e were from industrial processes and energy production [1]. Many economies, both developed and developing, rely heavily on fossil fuel combustion for heating and energy needs. Decommissioning CO_2_ emitting power plants to reduce carbon foot-print may not be a viable solution for many economies, especially for the developing economies. In recent years, there has been a concerted push to reduce CO_2_ emissions by using both conventional decarbonization technologies and negative emission technologies [1]. Negative emission technologies such as bioenergy carbon capture and storage (BCCS) and direct air carbon capture and storage (DACCS) both rely on catalyst driven capture of CO_2_ from industrial emissions or directly from ambient air. Both carbon capture and sequestration (CCS) technologies have been predicted to remove around 0.5 to 5 GtCO_2_ per year with an estimated removal of up to 40 GtCO_2_ per year by the end of the century [1]. Although progress has been made in carbon capture and sequestration (CCS) technologies, numerous hurdles still remain in the large-scale implementation of these processes for CO_2_ removal. Current CCS technologies rely on processes such as scrubbing or amine-based carbon capture, both of which can be energy intensive and expensive due to the cost involved in amine solution regeneration [2]. In recent years, the use of enzymes as an alternative to amine-based CCS technologies has been extensively tested, but insufficient activity during temperature variations and lack of enzyme stability in harsh conditions impede the large-scale implementation of this highly viable technology [2].

Identified in all domains of life, carbonic anhydrases (CA) are ubiquitous metalloenzymes that catalyzes the interconversion of CO_2_ to bicarbonate. CA's play an important role in critical physiological functions such as pH homeostasis, photosynthesis, CO_2_ transport etc. [3]. Based on structural, active site, and metal coordination differences, several different groups of CA's have been identified. Of these, α-CA's are one of the most extensively studied group of CA's due to its high catalytic efficiency [4], pharmacological [5], and biotechnological relevance [3]. All α–CA's are metalloenzymes with zinc in the active site, coordinated via three histidine residues and substrate binding occurring at the hydrophobic patch close to the active site [6],[7]. The catalytic mechanism is identical in all α–CA's; the reaction begins with the hydration of CO_2_ to bicarbonate followed by the displacement of bicarbonate by a water molecule that directly coordinates with zinc (Zn-bound hydroxide) [8]. Regeneration of Zn-bound hydroxide occurs via the intra and intermolecular proton transport achieved by a series of hydrophilic amino acids in the active site cavity. The final step in the CO_2_ hydration process is the transfer of the proton to the bulk solvent by a conserved histidine residue [8],[9]. This two-step reaction is essential for the efficiency of CA's and made possible by a series of hydrophilic residues in the active site [8]. Recently, there has been an uptick in the identification and characterization of prokaryotic α–CA's [10]–[15] due to its potential in CCS and biomineralization processes [6],[16]. Although several CA's from extremophiles have been characterized [11],[12],[17], their use is limited in CCS technologies due to the inherent instability of naturally occurring CA's. This issue is being partially addressed by engineering naturally occurring CA's with improved thermochemical parameters [2],[16], with one study demonstrating successful pilot scale CCS with engineered CA in alkaline solution [2]. Given the recent success of engineered CA in pilot scale CCS process, CA's with broader thermochemical stability can greatly improve the efficiency of the current CCS process for future full-scale use.

Previously, we solved the structure and partially characterized an extremophile α–CA's from *P. profundum* and discussed its unique structural element involved in halophilic protein dimerization [18]. In this study, we show that PprCA is indeed a unique enzyme that exhibits a bimodal pH activity with peak activity seen under both acidic (pH 5) and alkaline (pH 11) conditions. Unsurprisingly, PprCA is inactivated at high temperatures but showed salt-dependent temperature tolerance. In addition, PprCA exhibited high activity even at 1.5 M salt (sodium chloride and sodium sulfate) concentrations. Our previous structural analysis revealed the presence of N-terminal cysteine residues (Cys 33 and Cys 186) implicated in structural stability of CA's. Treating PprCA with increasing concentrations of a reducing agent, dithiothreitol (DTT) resulted in a concentration-dependent decrease in the catalytic activity of PprCA. This decrease was reversed by treating reduced PprCA with an oxidizing agent (diamide) which resulted in a time-dependent increase in PprCA catalytic activity. Finally, we demonstrate that PprCA is capable of accelerating biomineralization of CO_2_ in the presence of a cation, under alkaline conditions.

## 2. Materials and methods

### 2.1. Heterologous expression and purification of PprCA

Cloning, over-expression and purification of PprCA was performed either as reported previously in Somalinga et al, 2016 [18] or by using the gravity-based purification method. In the gravity-based method, a pET28a plasmid carrying the *pprCA* gene was transformed into competent *E. coli* BL21(DE3) (NEB). The transformed cells were grown in terrific broth containing 35 µg/mL Kanamycin at 37 °C, 200 rpm, until the culture reached an OD_600_ of 0.6. The temperature was then reduced to 20 °C, and protein expression was induced by adding 0.5 mM Isopropyl-β-thiogalactoside (IPTG). After over-night protein expression, cells were harvested by centrifugation (4000 rpm, 20 min at 4 °C) and stored at –20 °C until use. PprCA was purified by resuspending the pellet in Buffer A (20 mM Tris pH 7.5, 500 mM NaCl, and 30 mM imidazole), and the cells lysis was carried out using sonication. Cell debris from the lysate was removed by centrifugation (30 min at 12,000 rpm at 4 °C), and cleared lysate was filtered through a 0.45 µm syringe filter before being loaded on to a 1 mL Ni-NTA agarose (Qiagen) gravity column that was previously equilibrated with Buffer A. The protein was eluted with Buffer A containing imidazole (250 mM to 1M gradient), and the eluted protein was buffer-exchanged in 20 mM Tris pH 7.5, 150 mM NaCl buffer over-night at 4 °C. The purified protein was concentrated using an Amicon Centrifugal Filter, MWCO 10 kDa (Millipore) and stored at 4 °C until use.

### 2.2. CO_2_ hydration assay

The PprCA CO_2_ hydration assay was carried out according to Wilbur and Anderson, 1948 [19]. CO_2_-saturated water was prepared by bubbling CO_2_ gas through 250 mL of ice-cold water for 1 hour. The assay was performed by adding 4 mL of CO_2_ saturated water to 6 mL of 20 mM Tris, pH 8.3 buffer containing 100 µL of PprCA and immediately recording the drop in pH from 8.3 to 6.3 using a pH meter (Fisher Scientific, USA). The time taken (in seconds) for the pH to change from 8.3 to 6.3 is recorded as T. Blank reactions were performed by adding 100 µL of buffer instead of protein. The time taken for pH change in blank reactions were noted as T_0_. Enzyme activity expressed in Wilbur-Anderson units (WAU) was calculated using the formula: (T-T_0_) (dilution factor)/(T) (volume of enzyme in mL).

### 2.3. Thermal, pH and salt stability of PprCA

Thermal stability of PprCA was assessed by incubating 0.5 mg/mL of PprCA in 20 mM Tris pH 7.5, 150 mM NaCl for 60 minutes at 4 °C, 10 °C, 20 °C, 30 °C, 40 °C and 50 °C. Residual activity was assayed at 4 °C using CO_2_ as the substrate. In order to determine the pH stability of PprCA, 0.5 mg/mL of protein was incubated at different pH (pH 2 to 12) for 1 hour at room temperature, and residual activity was assayed at 4 °C using CO_2_ as substrate. Buffers used to achieve different pH were; 0.1 M Glycine-HCl buffer for pH 2 to 3, 0.1 Sodium citrate buffer for pH 4 to 6, 0.1 M Tris-HCl buffer for pH 7 to 9 and 0.1M Glycine-NaOH buffer for pH 10 to 12. The effect of salts on CO_2_ hydration activity of PprCA was determined by incubating 0.5 mg/mL of protein at room temperature for 1 hour with various concentrations of potassium chloride (KCl), sodium chloride (NaCl), sodium sulfate (Na_2_SO_4_) and sodium nitrate (NaNO_3_). Salt dependent temperature stability was determined by incubating PprCA in 20 mM Tris pH 7.5 buffer containing 1 M NaCl or Na_2_SO_4_ at temperature ranging from 20 °C to 45 °C. CO_2_ hydration activity of PprCA was assayed at 4 °C using CO_2_ as substrate.

### 2.4. Effect of 1,4-Dithiotheritol (DTT) and diamide on PprCA activity

The effect of DTT and diamide on PprCA activity was tested by adding increasing concentrations of (0.5 mM to 5 mM) of DTT to 0.5 mg/mL of PprCA and incubating at room-temperature for 30 min. The activity of DTT treated PprCA was tested using CO_2_ as the substrate. The effect of diamide on PprCA was assessed by adding 8 mM of diamide to 0.5 mg/mL of 4 mM DTT treated PprCA and incubating at room-temperature for 150 minutes. A 100 µL sample was taken at 30-minute intervals to test the CO_2_ hydration activity of PprCA.

### 2.5. Calcium carbonate mineralization assay

The calcium carbonate (CaCO_3_) mineralization assay was performed according to Jo et al., 2013 [20]. Briefly, the assay was initiated by adding 0.5 mL of CO_2_-saturated water to a cuvette containing 450 µL of reaction buffer (1 M Tris pH 8.4, 20 mM CaCl_2_), 50 µL of sample (1 mg/mL concentration or 1 M Tris buffer, pH 8.4 as blank) and mixed thoroughly. Immediately after the addition of CO_2_-saturated water, the cuvette was sealed with parafilm to prevent the leakage of CO_2_. CaCO_3_ mineralization in the form of precipitation was monitored visually for 30 min at room-temperature (22 °C). Buffers were prepared according to Jo et al., 2013 [20] while CO_2_-saturated water was prepared by bubbling CO_2_ into ice-cold water for 1 h.

## 3. Results and discussion

### 3.1. PprCA exhibits maximal activity under both acidic and alkaline conditions

**Figure 1.**
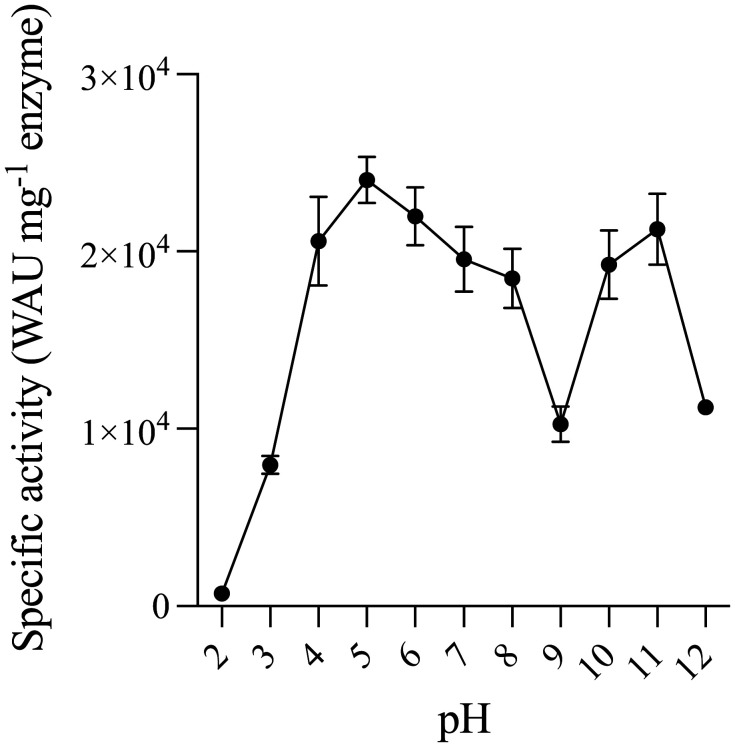
Effect of pH on PprCA activity. The pH maxima were ascertained using purified PprCA incubated for 1 hour at various pH in Glycine-HCl (pH 2 to 3), Sodium citrate (pH 4 to 6), Tris-HCl (pH 7 to 9) and Glycine-NaOH (pH 10 to 12) buffers. Residual activity of pH exposed PprCA was determined using Wilbur-Anderson assay. Values are means of 3 independent experiments, and the bars indicate standard deviation.

In order to determine the catalytic activity of PprCA, the protein was overexpressed in *E. coli*, and purified using immobilized metal affinity chromatography. The pH dependence of PprCA was determined at different pH (2 to 12) ranges using an electrometric method (Wilbur-Anderson assay). At extreme pH ranges (pH 2, 3 & 12), PprCA activity was negligible (Figure 1). On the other hand, PprCA showed high activity at pH 4 to 8 and pH 10 to 11 (Figure 1). Peak enzyme activity was observed at high alkaline pH (pH 11), and surprisingly even at acidic pH (pH 5), PprCA still retained 88% of its activity (Figure 1). To our knowledge, this type of dual pH activity at both alkaline and acidic pH has not been reported for any other prokaryotic α–CA's.

In *Chlamydomonas reinhardtii*, an α–CA, CrCAH3, is located in the thylakoid lumen and has been implicated in providing inorganic carbon for photosynthesis under low CO_2_ concentration conditions [21]. During photosynthesis, a light dependent acidification of the thylakoid lumen results in bicarbonate accumulation resulting in low photosystem II efficiency [21]. Conversion of bicarbonate to CO_2_ is catalyzed by CrCAH3 which shows optimal activity at acidic pH (~5.5) unlike its mammalian counterparts which show maximal catalytic activity close to or above neutral pH [22]. In bacteria, α–CA's have been predominantly localized to the periplasm and have been shown to be involved in physiological functions ranging from photosynthesis in cyanobacteria [23] to periplasmic pH homeostasis during acid acclimation in a Gram-negative human pathogen, *Helicobacter pylori* [24]. *H. pylori* α–CA is also capable of catalytic activity under acidic conditions, and CA activity has been proposed to be required for urea uptake and hydrolysis in this bacteria [25]. Although PprCA shows catalytic activity under both acidic and alkaline conditions, the physiological implications of this type of activity remains unexplored. We suspect that, similar to *H. pylori*, periplasmic localized α-CA in *P. profundum* may serve in maintaining pH homeostasis in this Gram-negative psychrophile.

### 3.2. PprCA N-terminal disulfide bond is required for structural stability

**Figure 2.**
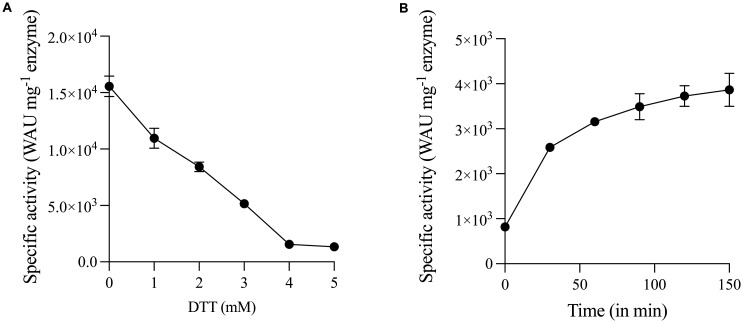
Redox conditions affect PprCA activity. The CO_2_ hydration activity of reducing (DTT) or oxidizing (Diamide) agent treated PprCA was determined using Wilbur-Anderson assay. A. Concentration-dependent reduction of CO_2_ hydration activity of PprCA treated with DTT (0.5 to 5 mM) for 30 min at room-temperature. B. Time-dependent increase in the CO_2_ hydration activity of PprCA treated with 4 mM DTT. Values are means of 3 independent experiments, and the bars indicated standard deviation.

The structure of PprCA was solved by Somalinga et al, 2016 [18], and revealed the presence of an N-terminal disulfide bond common in many well-characterized α-CA's. In order to ascertain the role of this disulfide bond, we subjected the purified PprCA to both reducing and oxidizing conditions. PprCA exposed to varying concentrations of DTT (0 to 5 mM) resulted in a 90% reduction in catalytic activity in a concentration-dependent manner (Figure 2A). At concentrations at and above 4 mM, PprCA retained only 10% of catalytic activity indicating the importance of the N-terminal disulfide bond. PprCA inactivated with DTT was used to ascertain the effect of oxidizing conditions on this protein. In a time-dependent manner, PprCA regained its catalytic activity under oxidizing conditions with diamide treatments that induced the formation of an N-terminal disulfide bond (Figure 2B).

**Figure 3.**
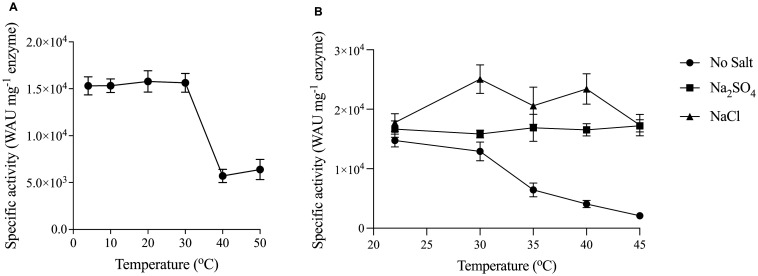
Effect of temperature and salt on thermostability of PprCA. A. Residual CO_2_ hydration activity of PprCA was determined after 60 min of incubation at the indicated temperatures. B. CO_2_ hydration activity of heat-treated PprCA in the presence of NaCl and Na_2_SO_4_. PprCA (0.5 mg/mL) was incubated at indicated temperatures in the presence of 1 M NaCl or Na_2_SO_4_ for 1 hour, and the residual activity was determined as indicated in Materials & methods. Values are means of 3 independent experiments, and the bars indicated standard deviation.

N-terminal disulfide bonds are a common feature in periplasmic localized α-CA's. In many of the well-characterized α-CA's, this disulfide bond is implicated in the structural stability and thermostability of the enzyme [10],[12],[14],[26]. In fact, given the importance of this disulfide interaction, studies have engineered this interaction into mesophilic counterparts to enhance structural and thermostability of the engineered enzymes [27],[28]. PprCA, similar to other characterized α-CA's, sports the N-terminal disulfide bond formed between Cys33 and Cys136 residues [18]; however, it does not enhance the thermostability of this enzyme (Figure 3A). In fact, our studies indicate that PprCA is inactivated at temperatures above 30 °C (Figure 3A). In addition to the terminal disulfide bonds, other factors such as having a compact, dimeric structure arrangement [26], and intermolecular disulfide bonds (12) have all been shown to play a role in the thermostability of α-carbonic anhydrases. While PprCA exhibits a compact structure, dimeric arrangement and an intramolecular disulfide bond [18], it lacks the thermostability of other similar α-CA's. This led us to surmise that the N-terminal disulfide bond may have function(s) other than imparting thermostability in PprCA.

In 2015, Benlloch et al. [22], characterized CrCAH3, a thylakoid lumen located, photosystem II membrane associated, α-CA from a green alga, *C. reinhardtii*. This protein contained all the hallmarks of intermembrane space targeted α-CA's including the terminal disulfide bond implicated in α-CA stability [22]. By probing the function of the CrCAH3 disulfide bond using DTT, a well-known reducing agent, the authors were able to show that CrCAH3 was inactivated even at low (0.5 mM) concentration of DTT. This inactivation was reversible, since CrCA3 activity was restored using an oxidizing agent in a time dependent manner [22]. This led the authors to conclude that the terminal disulfide bond in CrCA3 is involved in enzyme regulation, a conclusion supported in part because this type of regulatory mechanism is common in thylakoid luminal proteins involved in photosynthetic processes [29]. The structural, active site and disulfide bond position similarity between CrCAH3 and PprCA indicated that PprCA with its terminal disulfide bond may be involved in an enzyme regulatory mechanism akin to what is mediated by CrCAH3. Similar to results observed for CrCAH3, when PprCA was exposed to reducing conditions using varying DTT concentrations, a 90% reduction in catalytic activity with 4 mM DTT was seen (Figure 2A). It should be noted, however, that this concentration is 8 times greater than the concentration at which CrCAH3 lost almost 50% of its activity. PprCA's ability to withstand a high concentration of reducing agent is indicative of potential role in structural stability rather than having an enzyme regulatory function.

### 3.3. PprCA is inactivated at mesophilic temperatures, but its thermostability improves in the presence of salt

Thermostability is a coveted trait in enzymes that are designated for industrial use. Organisms that inhabit harsh environments have evolved mechanisms that can withstand extreme conditions. PprCA is expressed by *P. profundum*, a bacterium isolated from deep-sea sediment and classified as a moderately piezophilic, psychrohalophile [30]. Purified protein was used to determine the thermostability of PprCA at temperatures ranging from 4 °C to 50 °C. At low (4 °C to 20 °C) and mesophilic temperatures (30 °C), PprCA CO_2_ hydration activity was maximal and remained stable (Figure 3A). On the other hand, at temperatures above mesophilic range (>30 °C), PprCA showed a dramatic decrease in its catalytic activity (Figure 3A) even though it harbors the terminal disulfide bond implicated in the thermostability of other α-CA's. It is not surprising that PprCA is active only at psychrophilic and low mesophilic temperatures with decreased catalytic activity at temperatures at or above the mesophilic range. The simplest explanation is that because the optimal growth temperature for *P. profundum* is 15 °C, the enzymes from this bacterium have evolved to function optimally at psychrophilic temperatures. In addition to being a psychrophile, *P. profundum* is also a moderate halophile, a characteristic it shares with other members of the family Vibrionaceae.

In a 2015 study, Warden et al. [31], showed that a moderately halophilic bovine carbonic anhydrase II (bCAII) can be engineered to be extremely halophilic by manipulating surface amino acid residues in the protein. The authors employed a site-directed mutagenesis technique to mutate selected surface residues to acidic residues (aspartic acid and glutamate) and lowering the number for lysine residues in the protein. This resulted in an engineered bCAII with the ability to remain catalytically active at NaCl concentrations above 3 M and salt-induced stability at high temperatures [31].

PprCA is expressed in a bacterium that was isolated from deep-sea sediment where the salt concentration is approximately 600 mM which classifies *P. profundum* as a halophile. The work done by Warden et al., 2015 [31] with bCAII and the halophilic nature of *P. profundum* habitat prompted us to determine if high salt concentration improves thermostability of PprCA. Purified PprCA was incubated in 1 M NaCl and sodium sulfate (Na_2_SO_4_) at various temperatures for 1 hour, and residual activity was quantified using CO_2_ as a substrate. PprCA that was not exposed to salts was used as a control. In the absence of salt, PprCA exhibited activity only at psychrophilic and low mesophilic temperatures with drastic reduction in catalytic activity at mesophilic and thermophilic temperatures (Figure 3A and B). In contrast, PprCA exposed to NaCl (1 M) and Na_2_SO_4_ (1 M) retained 100% of its catalytic activity even at 45 °C indicating that certain salts improve thermostability of PprCA (Figure 3B). In engineered bCAII, Warden et al., 2015 [31] attributed the greater ability of the protein to withstand halophilic conditions to its increased surface exposed acidic amino acid residues and distribution of these residues across the surface of the protein. In addition, the authors also showed that engineered bCAII salt induced thermostability followed the Hofmeister series with high charge density anions such as sulfate and chloride contributing to its stability compared to cations [31]. PprCA, although not engineered, exhibits salt tolerance and salt-induced thermostability similar to engineered bCAII. The exact mechanism by which PprCA achieves halotolerance and salt-induced thermostability has not yet been investigated, but we suspect that it is similar to engineered bCAII and other characterized halotolerant α-CA such as dCAII from *Dunaliella salina* [32].

### 3.4. PprCA is inhibited by sodium nitrate and exhibits extreme halotolerance

**Figure 4.**
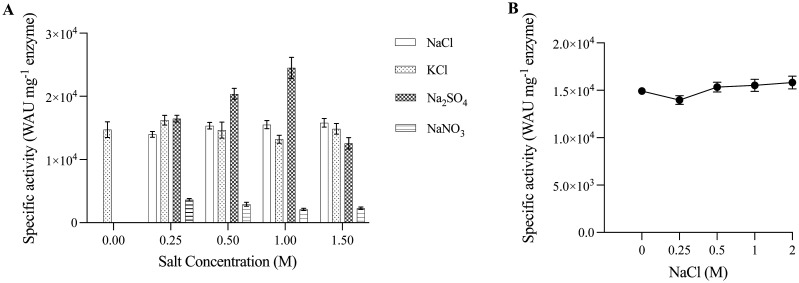
Effect of different salts and high NaCl concentration on the catalytic activity of PprCA. A. Residual CO_2_ hydration activity of salt-treated PprCA was ascertained by incubating the enzyme in buffer (20mM Tris pH 7.5) containing different salts (NaCl, Na_2_SO_4_, KCl or NaNO_3_) at concentrations ranging from 0.25 to 1.5M at room-temperature for 1 hour. B. CO_2_ hydration activity of PprCA exposed to NaCl concentrations ranging from 0.25 to 2M. All values are means of 3 independent experiments, and the bars indicated standard deviation.

Sodium chloride induced thermotolerance of PprCA (Figure 3B) was similar to other characterized extreme halotolerant α-CA's such as dCAI, dCAII, and engineered bCAII [17],[31],[32]. This property prompted us to investigate the effect of different salts on CO_2_ hydration activity of PprCA. Purified PprCA was incubated for 1 hour at 22 °C in 1 M NaCl, Na_2_SO_4,_ KCl or NaNO_3_, and CO_2_ hydration activity was measured using electrometric method as described above. As expected, catalytic activity of PprCA decreased in a concentration-dependent manner in the presence of NaNO_3_ (Figure 4A). Even at low NaNO_3_ (0.25 M) concentration, PprCA activity decreased by 70%, and at concentrations above 0.25 M, PprCA activity was reduced significantly (Figure 4A). In the presence of NaCl, PprCA remained catalytically active from concentrations ranging from 0.25 M to 2 M (Figure 4A and B), indicating that NaCl does not affect PprCA activity even at high concentrations. Finally, in the presence of Na_2_SO_4,_ CO_2_ hydration activity of PprCA increased in a concentration-dependent manner with peak activity seen at 1 M (Figure 4A). At a 1 M Na_2_SO_4_ concentration, catalytic activity of PprCA was ~50% higher than PprCA treated with NaCl and KCl (Figure 4A), although, at higher concentrations (1.5 M), catalytic activity decreased to untreated PprCA activity levels (Figure 4A).

Although CA's are one of the most extensively studied enzymes, to date, the number of halotolerant CA's characterized from both eukaryotic and prokaryotic sources remain limited. Two of the well-characterized halotolerant α-CA's, dCAI [17] and dCAII, are from a halophilic microalgae, *Dunaliella salina* [32]. dCA1, dCAII, and the engineered bCAII all exhibit halotolerance and enhanced salt-induced thermostability due to a set of characteristics unique to these enzymes that are not found in their mesophilic counterparts. These characteristics include, increased surface negative charge, reduced lysine and cysteine content, and higher small hydrophobic amino acid content [31]. In addition, ions such as Cl^−^, K^+^, and Na^+^ have been implicated in structural and inter-subunit stability of halophilic and thermophilic proteins [32]. In fact, crystal structure of PprCA showed a unique Cl^−^ ion in the dimer interface, which has been suggested to be involved in halophilic adaptation of PprCA [18]. Our work here also indicates that PprCA can tolerate hypersaline conditions akin to the engineered bovine α-CA, bCAII [31] and the α-CA's from *D. salina* [17],[32]. Similarly, in dCAII, unique Na^+^ ion bound surface α-helices have been implicated in the stability of this protein under halophilic conditions [32]. In addition to imparting structural stability, anions and cations are well known for their role in modulating CA activity. High charge density anions that share sodium as a cation, such as sulfate and chloride ions, have been shown to increase the catalytic activity of halophilic CA's [17],[31],[32]. In fact, PprCA's catalytic activity remained unaltered even at high NaCl (Figure 4A and B) and Na_2_SO_4_ concentrations (Figure 4A), mirroring the properties of other halophilic α-CA's. Monovalent anions are well-known inhibitors of CA activity. As expected, CO_2_ hydration activity of PprCA decreased dramatically even at low concentrations of NaNO_3_ (Figure 4A), a potent inhibitor of CA activity. Anions achieve this inhibition through interacting with the catalytically essential active-site Zn ion by disrupting Zn-hydroxide formation and by interfering with catalytically important water molecules in the active site [33].

### 3.5. PprCA accelerates CO_2_ biomineralization

**Figure 5.**
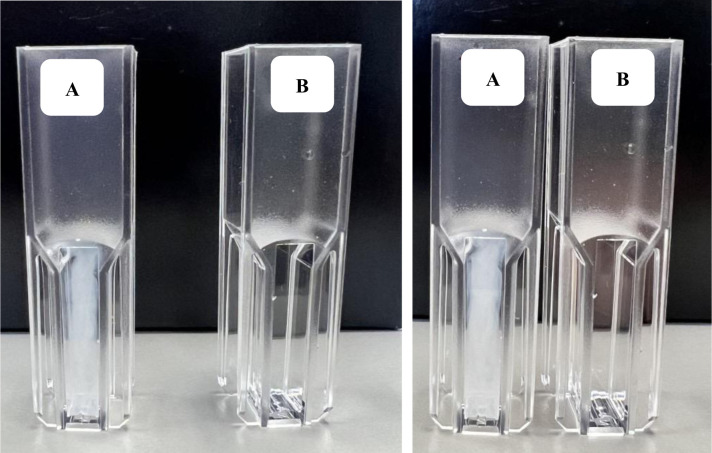
PprCA enhances CO_2_ biomineralization process. CO_2_ biomineralization to carbonates was determined qualitatively by visually examining for turbidity in reactions containing PprCA. Reactions were initiated by adding substrate (0.5 mL CO_2_ saturated water) to the reaction buffer (1 M Tris pH 8.4, 20 mM CaCl_2_) containing PprCA (1 mg/mL) and incubated at room-temperature for 30 minutes. Time-dependent increase in turbidity was observed in cuvettes (labeled A) containing PprCA, substrate and CaCl_2_. There was no observable turbidity in negative controls (cuvettes labeled B) without PprCA. Panel on the left shows reaction after 15 min of incubation and panel on the right shows reaction after 30 min of incubation.

CA's are one of the few enzymes that exhibit very high catalytic rates limited only by diffusion. High catalytic rates along with the ability to sequester CO_2_ efficiently have attracted interest in utilizing CA in industrial technologies ranging from carbon capture to production of value-added chemicals [34]. The unique biochemical characteristics of PprCA prompted us to explore its ability to mineralize CO_2_ in the presence of divalent cations such as Ca^2+^. Enzyme accelerated CO_2_ mineralization to calcium carbonate was assessed using purified PprCA in the presence of calcium chloride under alkaline conditions (pH 8.4). The reaction was initiated by adding substrate (CO_2_-saturated water) to the reaction mix and monitoring calcium carbonate precipitation at room temperature for 30 min. In the presence of PprCA, CO_2_ biomineralization to CaCO_3_ progressed rapidly as indicated by the appearance of turbidity in the solution (Figure 5, cuvettes labeled A). In the absence of PprCA, no turbidity was observed even after 30 min of incubation at room temperature indicating the enzymatic acceleration of CO_2_ biomineralization (Figure 5, cuvettes labeled B). CA-mediated carbonate precipitation is a well-characterized mechanism that requires alkaline pH, dissolved inorganic carbon, nucleation sites and cations such as Ca^2+^ [35]. Biomineralization of CaCO_3_ results in the formation of carbonate polymorphs including the stable calcite crystals and the metastable vaterite crystals [35]. Appearance of turbidity in reactions containing PprCA (Figure 5, cuvettes labeled A) indicated the rapid mineralization of CO_2_ into CaCO_3_ under controlled conditions. Although turbidity was observed, the nature of precipitate formed by PprCA induced biomineralization is still unclear. Investigation of the nature of the CaCO_3_ precipitate using high-resolution microscopic technique is ongoing.

## 4. Conclusions

The work presented here clearly demonstrates that PprCA exhibits several interesting biochemical properties such as salt-dependent thermotolerance, extreme halotolerance, and the ability to catalyze CO_2_ hydration in both acidic and alkaline pH. In addition, this work also reveals that PprCA is capable of accelerating CO_2_ biomineralization in the presence of Ca^2+^ ions under alkaline conditions. CA's that are capable of prolonged catalysis under operational extremes are sought after in industrial processes such as carbon capture. PprCA, although lacking in thermotolerance, exhibits other properties that make it an interesting candidate for CCS technologies. Protein engineering techniques can be utilized to improve PprCA's thermotolerance thereby making it attractive for industrial and direct CCS technologies.
